# 212 Urine Culture Stewardship Interventions in Hospitalized Adults with Indwelling Urinary Catheters: A Scoping Review

**DOI:** 10.1017/ash.2026.10596

**Published:** 2026-06-23

**Authors:** Sarah Yi, Qunna Li, Lacey Critchley, Lu Meng, Rahsaan Overton, Ibironke Apata, Nicole Gualandi, Jonathan Edwards, Jeneita Bell, Shannon Novosad, Matthew Stuckey

**Affiliations:** 1 Centers for Disease Control and Prevention; 2 General Dynamics Information Technology; 3 CDC; 4 Division of Healthcare Quality Promotion, NCEZID, CDC

## Abstract

**Background:** Resilient healthcare systems prevent, absorb, and learn from stressors. Identifying resilient performance in healthcare systems can be challenging due to complex underlying processes and associated outcomes. This study aimed to identify resilient performance in US outpatient hemodialysis (HD) facilities during the COVID-19 pandemic by characterizing associated operational factors and classifying longitudinal patterns in bloodstream infections (BSI) rates using machine learning. **Methods:** This study used longitudinal BSI data reported to National Healthcare Safety Network (NHSN) by outpatient HD facilities during pre-pandemic (April 1, 2018–April 30, 2019) and pandemic (April 1, 2021–April 30, 2023) periods. For each period, facilities were classified into distinct patterns based on facility-level BSI rates (cases per 100 patient months) using k-means clustering for longitudinal data (KmL), an unsupervised machine-learning method. CMS Dialysis Facility data provided key facility characteristics and NHSN Annual Dialysis Facility Surveys provided use of Core Interventions for Dialysis BSI Prevention. Facility resilience was operationalized as classification in a lower BSI rate cluster during the pandemic period. Associations between KmL classification during the pandemic and facility operational factors were assessed using multivariable logistic regression. **Result:** Of 7084 outpatient HD facilities, 4907 (69%) reported BSIs to NHSN during both periods and linked to CMS data. KmL grouped facilities into two clusters for each period. During the pandemic, a lower-rate cluster (n=4132, 84%) and a higher rate cluster (n=775, 16%) had mean BSI rates of 0.19 and 0.76 per 100 patient-months, respectively. Both clusters reported strong implementation of Core Interventions; however, the higher-rate cluster had increased odds of implementing ≤64% of interventions. In the multivariable model, facilities in the higher-rate pandemic cluster were associated with being in the higher-rate pre-pandemic cluster, structural factors (non-profit ownership, non-chain facility status, having <20 dialysis stations), processes (increased central vascular catheter use, lower use of antiseptic-impregnated catheter end caps, rarely administering antibiotics before obtaining blood cultures for suspected BSIs), and geography (Northeast or Midwest US location, rurality). **Conclusion:** Using machine learning, two distinct BSI rate trajectories emerged before and during the COVID-19 pandemic, with key operational differences between facility groups. Facilities demonstrating lower and stable BSI trajectories during pandemic could be interpreted as exhibiting more resilient performance under system stressors. This approach can inform resilient performance across other patient safety processes, healthcare settings, and system stressors. Understanding structures and processes associated with patient safety outcomes during disruption can support public health prioritization and targeted interventions to strengthen healthcare system resilience.

